# Paralinguistic Features Communicated through Voice can Affect Appraisals of Confidence and Evaluative Judgments

**DOI:** 10.1007/s10919-021-00374-2

**Published:** 2021-07-06

**Authors:** Joshua J. Guyer, Pablo Briñol, Thomas I. Vaughan-Johnston, Leandre R. Fabrigar, Lorena Moreno, Richard E. Petty

**Affiliations:** 1 Department of Social Psychology and Methodology, Universidad Autónoma de Madrid, Madrid, Spain; 2 Department of Psychology, Queen’s University, Kingston, Canada; 3 Department of Psychology, Durham University, Durham, UK; 4 Department of Psychology, The Ohio State University, Columbus, USA

**Keywords:** Vocal pitch, Emotional expressions, Confidence, Attitudes, Persuasion

## Abstract

This article unpacks the basic mechanisms by which paralinguistic features communicated through the voice can affect evaluative judgments and persuasion. Special emphasis is placed on exploring the rapidly emerging literature on vocal features linked to appraisals of confidence (e.g., vocal pitch, intonation, speech rate, loudness, etc.), and their subsequent impact on information processing and meta-cognitive processes of attitude change. The main goal of this review is to advance understanding of the different psychological processes by which paralinguistic markers of confidence can affect attitude change, specifying the conditions under which they are more likely to operate. In sum, we highlight the importance of considering basic mechanisms of attitude change to predict when and why appraisals of paralinguistic markers of confidence can lead to more or less persuasion.

## Introduction

In the words of the eminent philosopher Friedrich Nietzsche (1844–1900), “We often refuse to accept an idea merely because the tone of voice in which it has been expressed is unsympathetic to us.” Although intuitively, the importance of this concept is well understood, comparatively little attention has been devoted to examining how paralinguistic markers of psychological states communicated through the voice can affect the process of attitude formation and change. The present review addresses this gap by examining how inferences of confidence linked to vocal features such as pitch can play a critical role in cognitive and meta-cognitive processes relevant to attitudes and persuasion.

## Voice as Information

The voice can be a powerful source of information because it often provides valuable insight into the emotional and cognitive states of the communicator (Bänziger et al., 2014; Harrigan et al., 2008; Johnson et al., 1986; Scherer, 2019; Scherer et al., 2001). For example, studies have shown that vocal expressions of emotion can reliably inform a listener that a person is angry, sad, bored, fearful, or happy, and that the ability to recognize and distinguish among these linguistic markers of emotion transcends language barriers (Guyer et al., 2017; Juslin & Laukka, 2003). As an illustration of this phenomena, a study by Pell et al. (2009) revealed that monolinguistic native Spanish speakers were able to accurately identify vocally expressed emotions when listening to recordings in which the speaker shared their native language as well as when listening to recordings of English, Germanic, and Arabic speakers.

However, research indicates that in-group members tend to more accurately recognize differences in meaning associated with vocal expressions of emotions relative to out-group members (Laukka & Elfenbein, 2020; Mandal, 2008; Scherer et al., 2011). Interestingly, research has shown that even brief vocal expressions of emotion (i.e., vocal bursts) can affect our social interactions by revealing our inner feelings in ways that are difficult to fake (Cowen et al., 2019; Simon-Thomas et al., 2009). This suggests that vocal expressions yield information that people perceive as a valid basis upon which to make judgments (i.e., inferences about what people judge to be our genuine intentions and truthful inner emotional states).

Importantly, emotional expressions embedded in vocal features convey information beyond the expresser’s feelings. For example, based on vocal features, people make inferences about a speaker’s traits (Guyer et al., 2018a; Pisanski & Bryant, 2019), social intentions (Fraccaro et al., 2011; Hughes et al., 2010, 2014; Leongómez et al., 2014; Pisanski et al., 2018), and appraisal of the situation (Gregory & Webster, 1996; Puts et al., 2006). Indeed, a wealth of research within psychology, communications, and linguistics has shown that listeners make a wide variety of inferences and judgments about people based on changes in their voice. According to these literatures, voice provides a wealth of information related to demographic characteristics of a person such as sex, age, and social status (e.g., Cheng et al., 2016; Ko et al., 2015), various features of personality, including pleasantness (Zuckerman & Miyake, 1993), benevolence (Brown et al., 1973), competence (Kreiman & Sidtis, 2011; Sorokowski et al., 2019), honesty and anxiety (Apple et al., 1979; Bond et al., 1987), indicators of power, such as authority (Sorokowski et al., 2019), physical size, dominance, and strength (e.g., Klofstad et al., 2015; Pisanski & Bryant, 2019; Puts et al., 2006), credibility (Chebat et al., 2007; Gelinas-Chebat & Chebat, 1992, 1999; Smith & Shaffer, 1995), emotion (e.g., Andreasen, 1981; Halberstadt, 1983), attractiveness (e.g., Babel et al., 2014; Chattopadhyay et al., 2003; Feinberg, 2008; Hughes et al., 2014; Pisanski & Feinberg, 2019; Puts, 2016), attitude (e.g., Pittam & Gallois, 1987; Scherer, 1988), and the perceived persuasiveness of the speaker (Brooke and Ng, 1986; Hall, 1980; Mehrabian & Williams, 1969; Van Zant & Berger, 2020). Taken together, these data suggest that one important feature of oral communication is that voice conveys a rich variety of information beyond the content of a message.

One strategy by which people orally communicate their intentions, thoughts, and attitudes to others is through modulating the acoustic properties of their voice (Knapp et al., 2014; Schroeder & Epley, 2015, 2016). This practice occurs across a variety of contexts and is often motivated by a desire for social approval (Sorokowski et al., 2019). For example, research by Leongómez et al. (2017) explored this phenomenon within a professional context (i.e., job interview), demonstrating that interviewees raised their pitch when interacting with employers perceived as dominant and prestigious. The act of raising one’s pitch has been shown to reflect an attempt to indicate physical and/or social subordinance, in this case representing an acknowledgment of the social hierarchy between employer and job applicant.

Additionally, research has shown that people engage in vocal modulation to signal romantic interest in a prospective partner (Fraccaro et al., 2011; Leongómez et al., 2014; Pisanski et al., 2018). Within the context of speed dating, this behavior has been shown to occur for both males and females, such that men lowered their pitch and women raised their pitch to a greater degree when they were interested in a prospective romantic partner. In both cases, modulating one’s pitch led to more successful outcomes, suggesting that stereotypical associations between gender and pitch play an important role within the context of romantic interactions (Pisanski et al., 2018). Taken together, these studies suggest that not only do individuals intentionally modulate their vocal expressions, believing that this strategy will help to achieve a desired outcome, but they also extract useful pieces of information from the vocal cues embedded in others’ oral expressions, which in turn may inform their own attitudes (Mehrabian & Ferris, 1967).

Although many characteristics of voice could influence attitudes and persuasion, a growing body of research suggests one characteristic that should play an important role is the extent to which a speaker sounds confident (Brennan & Williams, 1995; Brown et al., 1985; Guyer et al., 2018a; Jiang & Pell, 2014; Kimble & Seidel, 1991; Scherer et al., 1973; Smith & Clark, 1993; Van Zant & Berger, 2020). Given that confidence is an important dimension people use to evaluate their own attitudes and thoughts (e.g., Briñol & Petty, 2009; Rucker et al., 2014), it makes sense that confidence should also be an important dimension people use when evaluating other’s communications. Indeed, recent work suggests that individuals preferentially dedicate attentional resources to detecting vocal signals that reflect varying degrees of confidence (Jiang & Pell, 2015, 2016). Moreover, appraisals of confidence can also be used to infer other attributes of the speaker, such as intelligence, expertise, knowledge, and social credibility (Guyer et al., 2018a; Pell, 2006; Scherer et al., 1973). Thus, to the extent that people infer confidence based on changes in voice and rely on confidence as a valid basis for making decisions, vocal confidence should be an important determinant of whether a persuasive appeal will successfully influence attitudes and behavior.

## Vocal Production and Vocal Perception are Tied to Speaker Confidence

Although the study of paralinguistic markers of vocal confidence has received limited attention within the persuasion literature, a diverse range of research within the domain of communications has documented which vocal characteristics vary according to a speaker’s confidence. Typically, this research has been conducted in several different ways. For instance, participants are sometimes explicitly instructed to speak in a confident versus unconfident manner, after which researchers have measured both perceived and actual changes in different characteristics of the speaker’s voice (e.g., vocal perception and vocal production; Jiang & Pell, 2015, 2017; Scherer et al., 1973). In other work, people have been observed in naturalistic settings where self-reports of confidence are typically either high or low (e.g., an authority figure giving instructions to others, versus untrained public speakers). Other research has manipulated people’s subjective ratings of confidence through experimental materials to observe how voice changes as a result of more naturally occurring confidence (e.g., vocal perception; Brennan & Williams, 1995; Kimble & Seidel, 1991; Smith & Clark, 1993). These methodologies have produced converging evidence indicating that specific variations in certain characteristics of voice systematically covary based on the extent to which a speaker is confident.

For example, several experiments have demonstrated that confident speakers tend to intentionally communicate at an objectively louder volume relative to unconfident speakers (Jiang & Pell, 2017; Kimble & Seidel, 1991; Scherer et al., 1973; Van Zant & Berger, 2020). Early research on vocal perception by Scherer et al. (1973) illustrated the relationship between vocal loudness and perceived confidence by instructing speakers to read a passage using either a confident or unconfident voice. The results indicated that speakers instructed to speak in a confident voice naturally spoke louder, faster, and with fewer pauses. Work by Jiang and Pell (2017) examined both vocal production and vocal perception, and found that speakers who were asked to communicate in a confident manner not only spoke at a louder volume, as revealed by subsequent acoustic analyses of the audio recordings (objective measures), but were also perceived as more confident by listeners (subjective measures).

Research indicates that changes in vocal intonation also vary as a function of speaker confidence (vocal production), as well as influencing listeners’ perceptions of speaker confidence (vocal perception; Bollinger, 1978; Brennan & Williams, 1995; Guyer et al., 2018a; Smith & Clark, 1993). For example, an experiment by Smith and Clark (1993) showed that when participants felt they lacked background knowledge (as assessed by a measure of confidence in their judgments), they tended to speak about that topic with rising vs. falling intonation (vocal production). Similarly, work by Brennan and Williams (1995) revealed that, when verbally responding to multiple-choice trivia questions, participants used rising intonation twice as frequently as falling intonation when providing incorrect responses. Moreover, participants who used falling intonation at the end of their sentences were perceived by listeners as significantly more confident than those who used rising intonation (vocal production and vocal perception).

As with vocal loudness and vocal intonation, research on vocal production has found that speakers increased their rate of speech when asked to speak in a confident manner (Jiang & Pell, 2014, 2017; Scherer et al., 1973). In concert with these data, research on vocal perception has shown that speakers who talk faster are perceived as more confident. For example, Scherer et al. (1973) had an experienced drama student record a passage while speaking in either a confident or doubtful manner. Results indicated that the speaker who spoke in a confident manner was perceived as communicating significantly faster as well as with greater fluency relative to the speaker who spoke in a doubtful manner. Likewise, work by Brown et al. (1985) on vocal perception instructed a speaker to read a passage at either a relatively slow, normal, or fast rate of speed. In line with prior research on vocal perception, a linear increase in ratings of speaker confidence was observed alongside increases in speech rate, once again revealing a link between perceptions of confidence and rapid speech. Numerous studies have replicated this pattern, suggesting a consistent relationship between perceptions of speaker confidence and rate of speech (Guyer et al., 2018a; Jiang & Pell, 2015, 2017; Monetta et al., 2008; Van Zant & Berger, 2020).

Finally, work on vocal production has also examined the link between confident speakers and changes in vocal pitch, demonstrating that speakers tend to communicate with a higher pitch when vocalizing unconfident expressions (Jiang & Pell, 2017). These findings have been corroborated by research on vocal perception, which has shown that listeners associated raised pitch with decreased confidence (Guyer et al., 2018a; Jiang & Pell, 2015, 2017; Monetta et al., 2008). For example, several experiments conducted by Guyer et al., (2018a) digitally manipulated the speaker’s vocal pitch to be either high or low, then evaluated the effect of this manipulation on participants’ ratings of speaker confidence and their attitudes toward various topics. As predicted, low pitch elicited significantly higher ratings of speaker confidence than high pitch. Moreover, low pitch also elicited more persuasion than high pitch.

Taken together, these methodologies have produced converging evidence demonstrating that relative to unconfident speakers, confident speakers speak faster, louder, use falling intonation at the end of their sentences, and have lower-pitched voices, and these same differences translate into higher perceptions of confidence. As further explained below, besides increasing perceptions of speaker confidence, it is important to note that these vocal qualities can also affect the impact of persuasive communication in several different ways. As explained shortly, in accord with prominent theories of persuasion, indicators of vocal confidence can affect: (a) the amount that people thoughtfully process a message, (b) the favorability of thoughts toward an advocacy, (c) the impact of generated thoughts on attitudes through a thought-validation process (metacognition), and also (d) the consequences (e.g., behavior) associated with attitude change. Before discussing these processes further, we next review a key vocal dimension associated with perceptions of confidence—vocal pitch frequency—and its impact on attitudes and persuasion.

## Vocal Pitch: The Nature of Fundamental Frequency

Of all the vocal hallmarks shown to reflect speaker confidence, a considerable amount of work within the domains of biology, physiology, and psychology has been devoted to better understanding vocal pitch. Research indicates that pitch is the most perceptually salient vocal property (e.g., Titze, 1994). In fact, these literatures suggest that the inferences a recipient makes about a target based on modulations in their vocal pitch (as opposed to modulations in other vocal properties) are uniquely linked to biological origins in both human and non-human primates (e.g., see Aung & Puts, 2020; Evans et al., 2008; Klofstad et al., 2015; Sorokowski et al., 2019; Taylor & Reby, 2010). Pitch refers to the subjective variation in the “highness” or “lowness” of voice resulting from differences in the fundamental vibration frequency (*F*_0_, measured in Hertz) caused by the length, tension, and cross-sectional area of the vocal folds in the larynx (Lieberman & Blumstein, 1988; Titze, 1994). Lower frequency vocalizations are often associated with males versus females, are linked with more facial hair, body size, strength, muscularity, and dominance (see Pisanski & Bryant, 2019 for a review), and are produced by many primate species to signal aggression and threat (Taylor & Reby, 2010).

Research indicates that the physical attributes typically linked to low pitch (i.e., size and strength) may have contributed to the emergence of pitch as a dominance cue (e.g., Wolff & Puts, 2010). In line with this, a variety of studies have found that a lower fundamental frequency is reliably associated with heightened levels of testosterone (Dabbs & Mallinger, 1999; Evans et al., 2008; Harries et al., 1997; Meuser & Nieschlag, 1977; Pedersen et al., 1986), which research has shown often serves as a marker of increased aggression and physical dominance among males (Mazur & Booth, 1998; Schaal et al., 1996; Swaddle & Reierson, 2002; Tremblay et al., 1997). Indeed, several studies have shown that males who perceived themselves as more socially dominant lowered their vocal pitch in response to mate competition, whereas the opposite pattern was found among males who perceived themselves as less socially dominant (Gregory & Webster, 1996; Puts et al., 2006).

Deep voices are also correlated with evolutionary success. Deep-voiced men are judged as more attractive by women (e.g., Feinberg et al., 2005b), mate more frequently (Hodges-Simeon et al., 2011), and father more children (Apicella et al., 2007). Such success has been attributed to perceptions of deep-voiced males as stronger (Feinberg et al., 2005a; Puts et al., 2012; Sell et al., 2010), as well as more physically and socially dominant (Puts et al., 2006, 2007; Wolff & Puts, 2010). Furthermore, recent work suggests that perceivers can accurately gauge upper body strength based on vocal pitch, a feature also used to infer fighting ability (Sell et al., 2010).

In a professional context, both men and women with relatively low vocal frequencies are typically judged as more dominant and competent (see e.g., Klofstad et al., 2012), and are more likely to be hired following a job interview (Schroeder & Epley, 2015). In fact, several studies have shown that voters prefer political candidates with lower-pitched voices (Anderson & Klofstad, 2012; Gregory & Gallagher, 2002; Klofstad et al., 2012; Tigue et al., 2012). Thus, a low-frequency voice may benefit men in a broad array of social contexts ranging from sexual to political and economic. This is not always the case among women, for whom low voice frequencies are also perceived as masculine (Pisanski & Feinberg, 2019) but can be considered unattractive (Feinberg, 2008; Puts, 2016).

Given the numerous advantages conferred by pitch across a variety of contexts, it makes sense that individuals with lower frequency voices tend to perceive themselves, and be perceived by others, as relatively more confident than individuals with higher frequency voices. Because pitch has a powerful influence on perceptions of speaker confidence (Guyer et al., 2018a; Jiang & Pell, 2015, 2017; Monetta et al., 2008), and people are more likely to behave in ways that are congruent with confidently held attitudes (Petty et al., 2007; Petty & Krosnick, 1995; Rucker et al., 2014), changes in vocal pitch should be an important determinant when evaluating whether a persuasive appeal will successfully influence attitudes and behavior.

## Understanding the Effects of Paralinguistic Markers of Confidence on Persuasion: A Theoretical Framework

Although a growing body of work has shown that changes in specific parameters of voice reliably influence perceptions of speaker confidence, comparatively little research has investigated the underlying mechanisms by which specific indicators of vocal confidence affect the degree of persuasion. Inconsistent results across studies have led some researchers to conclude that qualities of voice may enhance persuasion by serving as simple cues that affect perceptions of speaker credibility (e.g., Miller et al., 1976; Smith & Shaffer, 1995), whereas others propose its effects are likely driven by affecting how much people think about the message (e.g., Hausknecht & Moore, 1986; Moore et al., 1986; Smith & Shaffer, 1991). Although these inconsistencies may in part be attributed to methodological issues, a major problem facing this emerging literature is the absence of a general theoretical framework that can aid researchers by guiding their predictions regarding when (i.e., under what conditions) and why (i.e., by what processes) pitch and other vocal hallmarks of confidence affect persuasion.

**Fig. 1:**
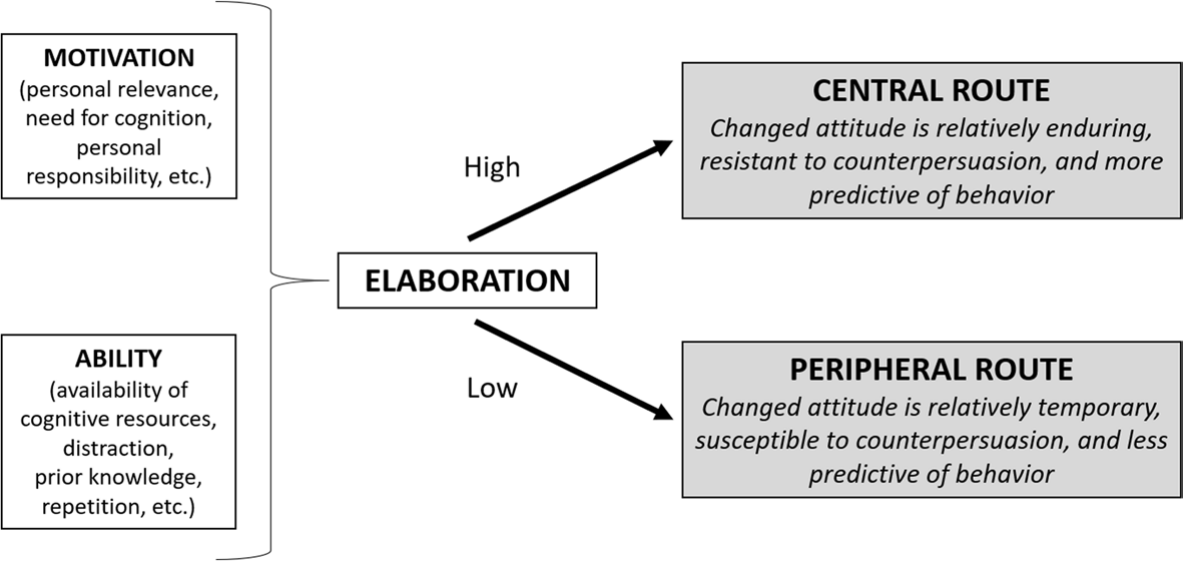
Schematic depiction of the Elaboration Likelihood Model. Adapted from Petty and Cacioppo (1986a)

In an attempt to reconcile these conflicting findings, recent work has drawn upon a prominent theory of persuasion known as the Elaboration Likelihood Model (ELM; Petty & Briñol, 2012; Petty & Cacioppo, 1986b; Petty & Wegener, 1998). The ELM is a general conceptual framework that describes a discrete set of psychological processes by which a given variable (e.g., vocal pitch) can produce different effects on attitudes according to specific conditions (influenced by contextual and dispositional factors), and also predicts the strength of the attitudes/evaluative judgments resulting from these processes. Which specific process emerges is determined by where a person falls on the elaboration continuum (i.e., from low-elaboration to high-elaboration), a construct which reflects the extent to which a person is motivated or enabled by individual and situational factors to think carefully about the information in a persuasive message (see Fig. 1). When ability and motivation to think are high, people tend to carefully examine the quality of the evidence provided (i.e., high-elaboration). In contrast, when ability and/or motivation are low, careful examination of the evidence is less likely (i.e., low-elaboration).

According to the ELM, at the low end of the elaboration continuum, a variable (e.g., paralinguistic markers of confidence such as vocal pitch) can influence attitudes by functioning as a simple peripheral cue, whereby evaluative judgments about a target may arise by way of a low thought process such as serving as a heuristic, classical conditioning, or a self-perception process (e.g., the message must be right because the speaker seems confident). At the high end of the elaboration continuum, a variable affects attitudes by a process that requires more thought. Thus, the variable can either (1) serve as an argument for or against the message, (2) bias the direction of thoughts to be more or less favorable, or (3) determine whether an individual relies on their own thoughts generated in response to a message (i.e., confidence / liking for one’s own thoughts). In the middle of the elaboration continuum, when processing is not constrained to be either high or low by other factors, a variable can affect the amount of processing that occurs. Each of these underlying mechanisms, known as *multiple roles*, are explained in more detail shortly.^1^

**Fig. 2:**
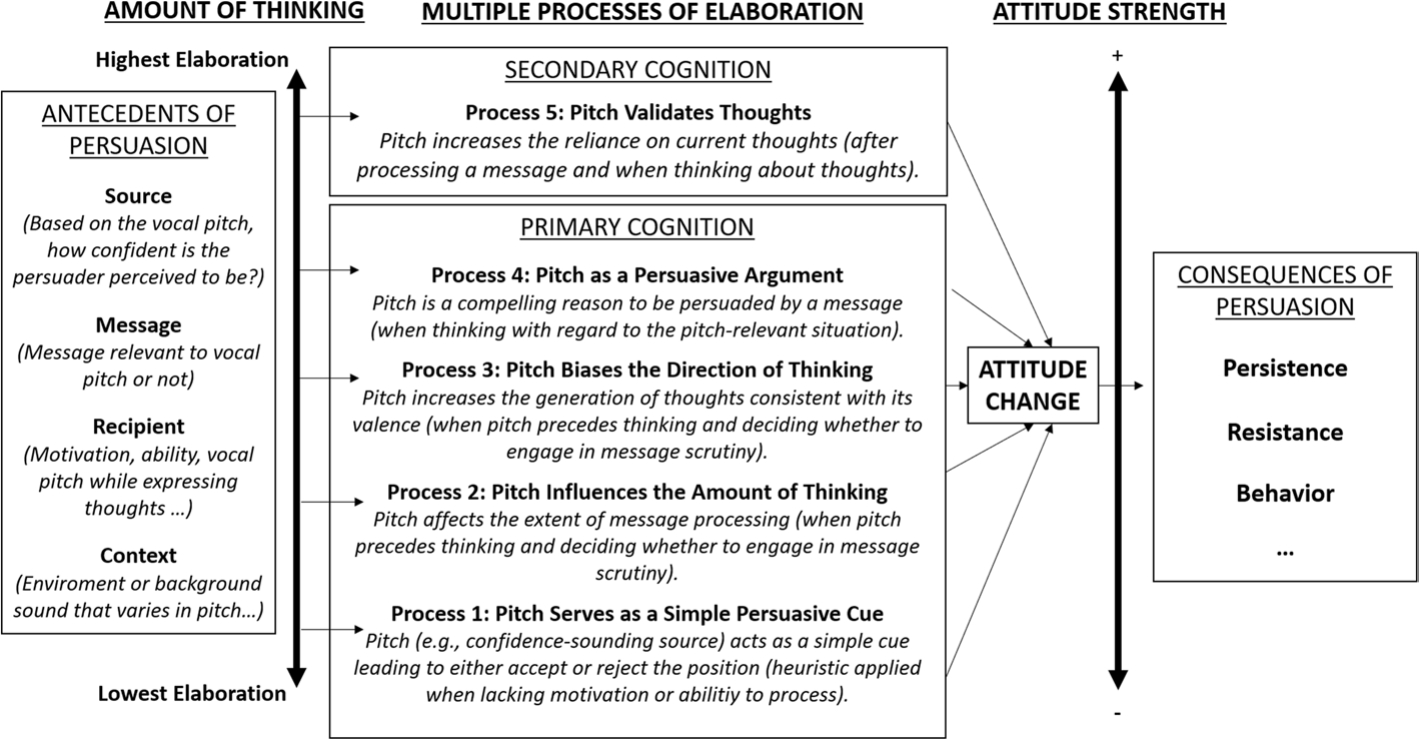
Main antecedents, processes, and consequences in the extended ELM (adapted from, Briñol et al., 2018). Although the figure focuses on pitch, the same processes apply to other vocal properties like intonation, speed, etc.

Importantly, the ELM states that whether attitudes are changed by processes associated with relatively high or low thinking has important downstream implications for the strength, durability, and resistance of the attitude. For example, as illustrated in Fig. 2, as the elaboration involved in attitude change increases, the resulting attitude typically becomes more persistent, resistant, and predictive of intentions and behaviors (Haugtvedt & Petty, 1992; Haugtvedt & Strathman, 1990; Petty et al., 1983, 1995a, 1995b). Moreover, the ELM explains how multiple processes of persuasion can operate in different circumstances. That is, the ELM proposes that any given variable can influence persuasion in different ways and therefore produce *different* outcomes. For example, whereas under low-elaboration conditions, a confident-sounding person can increase persuasion when their voice serves as a simple positive cue, under moderate-elaboration conditions, a confident-sounding person can decrease persuasion when their voice reduces careful thinking about strong arguments.

The ELM also indicates that *similar* outcomes can be produced by different mechanisms that operate at different places along the elaboration continuum (i.e., ranging from low to high thinking). For example, a confident-sounding person can increase persuasion when their voice serves as a simple positive cue (low-elaboration), when it decreases thinking about weak arguments or increases thinking about strong arguments (moderate-elaboration), when it serves as a strong argument itself (high-elaboration), when it biases the direction of thoughts that are generated to match the position advocated by the person or group delivering the message (high-elaboration), or when a speaker’s voice either validates positive thoughts that have already been generated or invalidates negative thoughts (high-elaboration). Importantly, as previously noted, the ELM postulates that not all judgmental outcomes that appear the same on the surface really are the same (e.g., attitudes changed to the same extent via high versus low-thinking processes are differentially persistent over time). As we illustrate in the following sections, knowing that a confident-sounding person can influence persuasion is not enough. Rather, it is also vital to understand the psychological process by which this apparently straightforward effect (or the contrary) occurs. Although many aspects of a persons’ vocal qualities can influence attitudes via these multiple processes, we highlight vocal pitch for illustrative purposes.

## Low Elaboration: Pitch can Influence Persuasion as a Peripheral Cue

One process by which vocal pitch can influence attitudes and persuasion is by operating as an indicator of the speaker’s confidence, in turn, serving as a simple cue leading the message recipient to either accept or reject the position advocated in a message when thinking is low. The impact of pitch under low elaboration conditions depends on whether the meaning associated with pitch is positive or negative. For example, a speaker whose vocal pitch is low because they are believed to have a cold or sore throat (negative meaning associated with low pitch), is unlikely to be perceived as more confident and thus more persuasive. Similarly, a speaker whose vocal pitch is high because they inhaled helium is unlikely to be perceived as unconfident and thus less persuasive. Low-elaboration conditions can occur by way of situational factors (e.g., distraction, low-involvement, low relevance/responsibility, etc.), and/or via dispositional factors such as low *need for cognition* (i.e., individuals who dislike cognitively demanding tasks; Cacioppo & Petty, 1982; see Petty & Wegener, 1998 for a review of variables that impact thinking).

In a series of experiments, Guyer et al., (2018a) examined the role of vocal pitch as an indicator of confidence, capable of serving as a peripheral cue to persuasion under low-thinking conditions. Participants heard a speaker whose vocal pitch was digitally manipulated to be either comparatively low or high. Additionally, participants were randomly assigned to either a high or low elaboration condition prior to receiving the audio passage. Specifically, high-thinking conditions were created by providing a semi-private environment free of all distractions, thus maximizing participants’ ability to thoughtfully process the message (Petty et al., 1976). Similarly, motivation to process the message was maximized by including a manipulation of personal responsibility, which research has shown can enhance motivation to process issue-relevant arguments (Petty et al., 1980). In contrast, low-elaboration conditions were created by way of a distraction task that required participants to memorize and later recall an eight-digit number, thus reducing their ability to carefully process information (e.g., Gilbert & Osborne, 1989). Likewise, motivation to thoughtfully evaluate the message was reduced by informing participants that their responses may be discarded and were unlikely to be read by the researchers. After listening to the audio recording, participants indicated their attitude towards the topic, then evaluated different attributes of the speaker, including the speaker’s perceived confidence. Lastly, participants listed and rated the valence of their thoughts (i.e., positive, negative, neutral, or unrelated; see Petty & Cacioppo, 1986a), as those thoughts applied to the persuasive proposal.

**Fig. 3:**
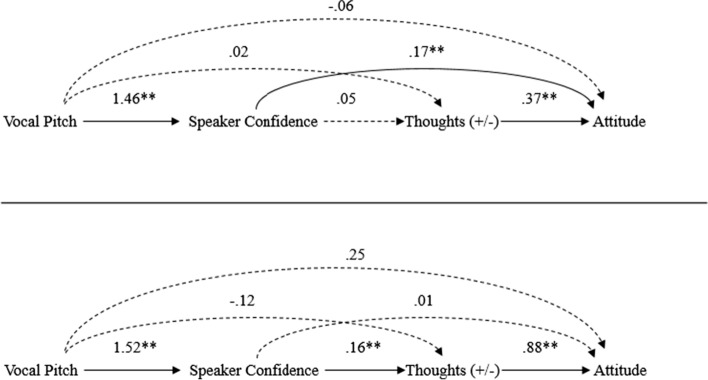
Top panel: The effects of vocal pitch on persuasion as a peripheral cue under low elaboration. Bottom panel: The effects of vocal pitch on persuasion as a biasing factor under high elaboration. Data from Guyer et al. (2018a)

Confirming expectations, changes in vocal pitch predicted ratings of speaker confidence, with low pitch receiving significantly higher ratings of confidence than high pitch within both high and low elaboration conditions. Importantly, under low elaboration, speaker confidence served as a peripheral cue by directly affecting participants’ attitudes, such that higher perceptions of speaker confidence led to more favorable attitudes *without* affecting thought favorability (i.e., a thought-biasing effect was not found), which is exactly what the ELM would predict under conditions that are not conducive to careful thinking (Petty et al., 1993). Thus, vocal pitch can increase perceived confidence, in turn, serving as a peripheral cue whereby listeners directly infer their attitudes based on the speaker’s apparent confidence (see Fig. 3, top panel). In depth coverage of research illustrating how different variables can influence attitudes as a peripheral cue can be found in Guyer et al. (2019). Next, we see how in this same study, pitch affected attitudes by a different mechanism under high elaboration.

## High Elaboration: Pitch can Influence Persuasion by Biasing Thoughts

Beyond serving as peripheral cue under low-thinking conditions, vocal pitch can also influence attitudes by different processes under different circumstances. For example, when a message recipient is able and motivated to carefully consider the merits of an issue (i.e., high-elaboration), a speaker’s vocal pitch can bias the valence/direction of a recipient’s thoughts in response to a persuasive message. The greater the room for interpreting information (e.g., when persuasive information is ambiguous), the more likely it is that paralinguistic markers of confidence like pitch will bias the direction of the thoughts generated, especially when multiple interpretations of the information are possible (Chaiken & Maheswaran, 1994).

Evidence for pitch biasing thoughts (see Fig. 3, bottom panel) was presented in the experiment described in the last section on peripheral cues by Guyer et al., (2018a). Specifically, consistent with the idea that vocal pitch can bias the direction of thinking about an issue, thoughts were significantly more positive when the speaker communicated using low (vs. high) pitch. Importantly, thought favorability significantly mediated the relationship between ratings of speaker confidence and recipient’s attitude toward the advertisement. Moreover, this pattern of effects was replicated in a follow-up study, thus providing further support that vocal pitch can serve in a biasing role under high-elaboration conditions. Research by Chattopadhyay et al. (2003) revealed a similar thought-biasing pattern under high-thinking conditions, in which low pitch led to more positive cognitive responses toward the topic, and cognitive responses mediated the relationship between pitch and attitudes toward the target.

In sum, we have thus far discussed how pitch can affect persuasion by serving as an indicator of confidence, in turn, functioning as a cue under low-elaboration conditions or by biasing thoughts under high-elaboration conditions. As previously noted, whether the process by which persuasion takes place involves low or high elaboration is important because the consequences associated with those changes are likely to be different. For example, research has shown that effortful processing of a message typically yields attitudes that are more accessible, durable over time, resistant to persuasive attacks, and more predictive of behavior than attitudes formed by relatively low-thinking processes (see Petty et al., 1995a, 1995b, for a review). Future research should examine to what extent the high and low-elaboration processes examined by Guyer et al., (2018a) for pitch might differentially impact the strength of resulting attitudes.

## High Elaboration: Pitch can Influence Persuasion as an Argument

In addition to biasing the direction of thoughts, vocal pitch can also serve as an argument either for or against an attitude object when this feature of a speaker’s voice is relevant to evaluating the merits of the advocacy and when thinking is high. For example, if one’s goal is to determine whether it would be good or bad to hire a person as a radio announcer or as a host for sporting events, then the properties of the person’s voice are likely an important piece of information or evidence relevant to evaluating their suitability for the role.

Consider an advertisement promoting a new program designed to improve public speaking. One aspect relevant to evaluating the effectiveness of a public speaking program is whether people who have taken the program speak with confidence. That is, a confident sounding speaker might be viewed as a relevant argument in favor of the program because a listener may reason that the program taught the speaker how to communicate with confidence. Likewise, a listener may evaluate an unconfident speaker as evidence suggesting that the program is ineffective at teaching people how to communicate with confidence. Importantly, the extent to which a speaker sounds confident when delivering a message can only serve as an argument when the speaker’s confidence is relevant to the advocacy. For instance, a speaker’s confidence would be relevant in the context of a message advocating a program designed to improve public speaking, but would be quite irrelevant in the context of a message advocating a program designed to improve one’s ability to take appealing photographs.

Recent work by Vaughan-Johnston et al. (2020) examined for the first time how a speaker’s vocal pitch and speech rate might affect a recipient’s attitude by serving as an argument either in favor of or against the position advocated in the message. Participants listened to an audio recording that described one of two program types—one designed to help them speak with confidence (i.e., a topic relevant to pitch/speech rate), versus one designed to help them take good pictures (i.e., a topic irrelevant to pitch/speech rate). For each topic, the authors created an audio recording in which the speaker’s vocal pitch and speech rate were both digitally manipulated within the same recording in order to create two conditions that either represented high or low speaker confidence (i.e., low pitch/fast speech vs. high pitch/slow speech). After listening to one of the four audio recordings, participants indicated their attitude toward the improvement program, provided a judgment of speaker confidence, and then completed a thought-listing task. Elaboration was globally set high by leading participants to believe that only a few people were taking part in the study, therefore their responses were especially important.

**Fig. 4:**
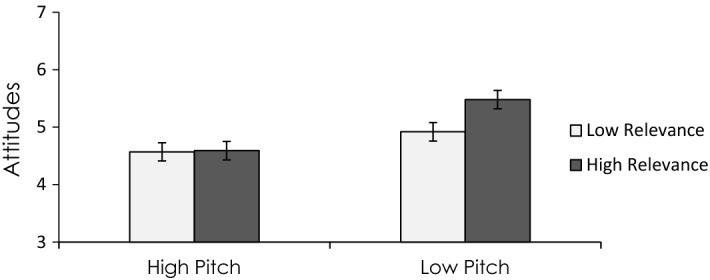
High elaboration: The effects of vocal pitch on persuasion as an argument. Data from Vaughan-Johnston et al. (2020)

As expected, the paralinguistically confident speaker was perceived as more confident than the paralinguistically unconfident speaker, independent of the type of program (i.e., public speaking vs. photography). Most importantly, a two-way interaction between the paralinguistic confidence condition and the program type showed that when perceived speaker confidence was relevant to evaluating the quality of a program (public speaking), speaking confidently (i.e., low pitch/fast speech) led to more positive attitudes toward the program than speaking unconfidently (i.e., high pitch/slow speech). However, when perceived speaker confidence was not relevant to evaluating the quality of a program (photography), then differences in paralinguistic confidence (low vs. high) had no effect on participant’s attitudes toward the program (see Fig. 4). This pattern of effects was replicated in a second experiment using a sample of native Spanish speakers (vs. Canadians speaking English in the study just described), thus providing some basis to suggest that this effect is replicable and generalizes across both language and culture. Illustrating the generalizability of this psychological process to variables beyond vocal pitch, recent research has shown that other variables beyond pitch (i.e., physical attractiveness) can also affect attitudes by serving as an argument (Kang & Herr, 2006; Mello et al., 2020).

## Moderate Elaboration: Pitch can Influence Persuasion Via Amount of Thinking

In certain situations, there may be a relative absence of factors that promote either high or low elaboration. Under such conditions, a message recipient’s ability and motivation to think can be described as *unconstrained* or free to vary as a function of various markers of the vocal confidence of the speaker (e.g., pitch, speech rate, intonation). That is, when no constraints are placed on a person’s ability and/or motivation to think, paralinguistic markers of confidence can influence attitudes by either increasing or decreasing the amount of thinking depending on the reactions they produce in the recipients of a message. One of the simplest ways of evaluating whether a variable affects processing is to determine the extent to which strong arguments are more persuasive than weak arguments when that variable is present rather than absent. Weak arguments are those advocating in favor of a proposal but, unlike strong arguments, weak arguments use reasons that are not compelling (e.g., people should vaccinate because the injections are delivered in colorful syringes). When people process weak arguments, they are more likely to recognize the lack of merits and generate counter-arguments (reducing persuasion). However, when people do not process weak arguments (e.g., due to the confidence produced by the speaker or by a distraction), they are less likely to counter-argue (they do not recognize the flaws), and therefore persuasion increases relative to when processing is high. Thus, variables that increase elaboration, such as personal relevance (Petty & Cacioppo, 1979) or personal responsibility (Petty et al., 1980), should increase the tendency of strong arguments to produce more persuasion than weak arguments, whereas variables that reduce elaboration, such as distraction (Petty et al., 1976) or time pressure (Gilbert & Osborne, 1989), should reduce the tendency of strong arguments to produce more persuasion than weak arguments. This is because when people think carefully, they tend to generate favorable thoughts to strong arguments, resulting in enhanced persuasion, but counter-arguments to weak arguments, resulting in opinions that contradict the message (see Petty & Wegener, 1998, and Carpenter, 2015, for reviews).

Consider how vocal pitch might influence persuasion when thinking is unconstrained (i.e., moderate elaboration). When people are uncertain whether to devote cognitive resources to processing a message, a relatively low pitch (confident) voice might enhance a recipient’s motivation to scrutinize the quality of the arguments in a message over a high pitch (non-confident) voice, thus increasing the impact of argument quality on attitudes. This is because a confident sounding person may be perceived to know what he or she is talking about, thereby justifying exerting effort to process the arguments because doing so would be worthwhile (Heesacker et al., 1983). Thus, when no constraints are placed on a person’s ability and motivation to think, vocal pitch can potentially influence the success of persuasive communications by affecting perceptions of speaker confidence, which can affect persuasion by influencing the extent of thinking about the message. Further examples of the effects of different variables on attitudes under moderate elaboration can be found in Guyer et al. (2019).

## High Elaboration: Pitch can Influence Persuasion Via Metacognition

As illustrated so far in this review, analyses of people’s attitudes have focused on how persuasion processes affect mostly the number (how many) and valence (positive or negative) of the thoughts people generate. That is, initial work on pitch and persuasion, like other vocal factors, has examined the nature or content of the primary cognitions that people have prior to a judgment. However, a large body of research now suggests that secondary (meta-cognitive) reflections are also important to consider (Briñol et al., 2012). For example, to what extent do people think their primary cognitions are valid?

The process of reflecting on the validity of one’s thoughts highlights the distinction between primary and secondary cognition. Primary cognition refers to thoughts that occur at a direct level and involve initial associations of some object with some attribute (e.g., this product seems good). However, following a primary thought, people can also generate other thoughts, which occur at a second level and involve reflection on their initial thoughts (e.g., I am confident that this product seems good). Metacognition refers to these second-order thoughts (thoughts about thoughts; for reviews, see Briñol & DeMarree, 2012; Dunlosky & Metcalfe, 2009; Jost et al., 1998; Petty et al., 2007). Therefore, under conditions in which careful scrutiny of a message is likely (i.e., high elaboration), the ELM proposes that attitude change can occur as a result of secondary cognition; for instance, via a *thought validation* process (Petty et al., 2002).

The key notion of thought validation is that the greater the perceived validity of one’s thoughts, the more those thoughts are translated into overall judgments. Thus, two people might have the very same thought, but one person might believe that the thought is more valid than the other person does, and is therefore more likely to form a judgment based on it and act upon it. People can rely on their thoughts because they believe the thought is correct (cognitive validation) or because they feel good about it (affective validation; Briñol et al., 2018). Meta-cognitive thoughts regarding the perceived validity of primary thoughts are important because such secondary thoughts can magnify, attenuate, or even reverse the impact of primary thoughts on judgment and action (Petty et al., 2007). Perceptions of validity are influenced by both situational and person variables alone and in combination, and their impact on judgment can vary with their meaning. Many variables arising from the situation have been shown to impact validation processes, ranging from source credibility to numerical status and power (Briñol & Petty, 2009). In this section, we focus on vocal pitch as a recently identified variable that can also influence persuasion by affecting thought validity under high-thinking conditions.

As an example of how a person’s vocal pitch can influence his or her own attitudes via meta-cognitive processes, consider recent work by Guyer et al. (2020). In this study, participants first read a passage discussing either the advantages or disadvantages of requiring senior comprehensive exams to complete one’s undergraduate degree. After reading the passage, participants were asked to list their thoughts about the topic and then rate the valence of their thoughts (i.e., using the thought listing technique). Next, participants were presented with the identical passage, but on this occasion, they heard the passage delivered by a speaker whose vocal pitch and speech rate were both digitally manipulated within the same audio recording in order to create two conditions that represented either high or low speaker confidence (low pitch/fast speech vs. high pitch/slow speech). Following this, participants rated the speaker on various dimensions, including confidence, then completed the thought listing task a second time, on this occasion indicating to what extent they were confident in their thoughts. Finally, a measure of participant’s attitude toward the exams was obtained.

**Fig. 5:**
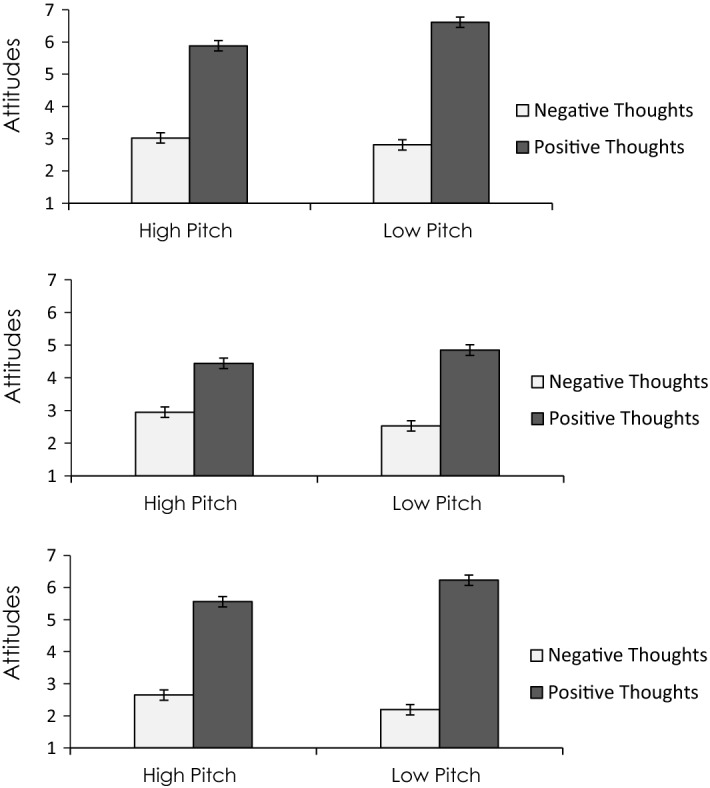
Top panel: The meta-cognitive effects of source vocal pitch on persuasion under high elaboration. Middle panel: The meta-cognitive effects of recipient vocal pitch on persuasion under high elaboration. Bottom panel: The meta-cognitive effects of pitch on persuasion as a context factor under high elaboration. All data are from Guyer et al. (2020)

As predicted, the speaker was perceived as more confident when communicating using low pitch/fast speech versus high pitch/slow speech. Of critical importance, a two-way interaction emerged between the paralinguistic confidence condition and the direction of participants’ thoughts on their subsequent attitudes toward the exam. Specifically, when the speaker expressed positive thoughts about the exam in a paralinguistically confident manner, this caused participants to report more positive attitudes than when the speaker expressed their positive thoughts in a paralinguistically unconfident manner. Conversely, when the speaker expressed their negative thoughts about the exam in a paralinguistically confident voice, this caused participants to report more negative attitudes than when the speaker expressed their negative thoughts in a paralinguistically unconfident voice (see Fig. 5, top panel). Mediation analyses confirmed that the speaker’s vocal pitch influenced recipient’s attitudes toward the topic based on how it affected recipient’s confidence in their own thoughts rather than the valence of their thoughts (which would have been the case if pitch increased processing of the message). Thus, this two-way interaction between thought direction and pitch was interpreted as a case in which speaker confidence (arising from low pitch) validated thoughts because thought confidence but not thought favorability mediated the effect. This research is consistent with recent research showing that pitch and other voice features can be manipulated to study their impact on the speaker (Arias et al., 2021). Further examples of research that illustrate the effect of other features of the speaker affecting attitudes via meta-cognitive validation of thoughts can be found in Briñol and Petty (2009).

Finally, it is important to note that if people believe that their judgments may have somehow been inappropriately biased by the properties of a communicator’ voice (e.g., vocal pitch), and they do not want this to occur, they can correct their judgments in a direction opposite to the perceived bias (i.e., engage in correction processes, Petty & Wegener, 1993; see Wegener & Petty, 1997, for a review). These meta-cognitive processes are most impactful when thinking is high because it is only under conditions of careful thought that people generate a substantial number of issue-relevant thoughts that carry the potential to shape their attitudes. Importantly, corrections to one’s thoughts can occur in different directions (i.e., in favor or against an advocacy) depending on recipients’ theories of how the biasing event or stimulus (e.g., a confident sounding speaker) influenced their thoughts. When people are motivated and able to correct, theory-based corrections can lead to reversals of typical persuasion effects (e.g., an unconfident speaker could be more persuasive than a confident speaker if a person “overcorrects” for the perceived influence; cf. Petty et al., 1998).

## The Metacognitive Role of Pitch Beyond Persuasive Sources

To this point, we have illustrated five psychological processes proposed by the ELM through which changes in vocal pitch originating from the source of a message can affect perceptions of speaker confidence and, in turn, influence persuasion via primary and secondary cognition. Recent work suggests that the link between pitch and confidence can arise from factors beyond a speaker’s voice, such as when the recipients themselves speak in a low/high pitch voice, or a low/high pitch sound is incidentally present in the environment.

Given that the confidence with which others vocally express their thoughts can impact the favorability of one’s own thoughts and attitudes via a meta-cognitive process, this raises the possibility that how people vocally express their own thoughts can also influence whether and how they use their thoughts to inform their attitudes. For example, consider a person who vocally expresses either their positive or negative thoughts using qualities of voice that reflect confidence (e.g., low pitch) versus doubt (e.g., high pitch). We might expect that expressing one’s positive thoughts in a confident manner should yield more positive target-relevant attitudes than expressing one’s positive thoughts in a doubtful manner. In a similar fashion, expressing one’s negative thoughts in a confident manner should yield more negative target-relevant attitudes than expressing one’s negative thoughts in a doubtful manner. In other words, confidently expressed thoughts should be more impactful in determining one’s attitudes.

This premise was tested for the first time in an experiment by Guyer et al. (2020) using a sample of Spanish-speaking undergraduate students. After reading a passage discussing either the advantages or disadvantages of comprehensive final exams, participants then listed up to three positive or negative thoughts based on the condition to which they were assigned. Next, all participants received a cover story ostensibly from the university IT department that asked them to help test the sound quality of a recently acquired audio recording program by reading aloud the thoughts they had previously written using either a higher or lower pitch than they would normally use when talking. Finally, participants reported their attitudes toward comprehensive exams, then viewed the thoughts they had previously written and indicated to what extent they were confident in each thought.

As predicted, the results showed that whereas vocally expressing positive thoughts using low versus high pitch led to more positive attitudes, vocally expressing negative thoughts using low versus high pitch led to more negative attitudes (see Fig. 5, middle panel). In other words, how people vocally expressed their thoughts influenced persuasion. Importantly, in line with the thought-validation hypothesis, these data suggest that the meta-cognitive effect of vocal pitch on attitudes was mediated by perceptions of confidence in one’s thoughts. Moreover, these data provide initial evidence suggesting that, in the same way as changes in vocal pitch influence how confident people perceive others to be, vocally expressing one’s own thoughts using high or low pitch can also influence perceptions of one’s own self-confidence.

A subsequent experiment by Guyer et al. (2020) tested whether the meta-cognitive effects of pitch on attitudes might also emerge when pitch functioned as a contextual variable. That is, whether subtly exposing a person to a low vs. high pitch sound occurring in the background while they generated thoughts might also yield a meta-cognitive effect on attitudes via thought-confidence. Once again, participants first read a passage describing either the advantages or disadvantages of comprehensive exams, then used a keyboard to type either positive or negative thoughts matching the valence of the passage. While participants typed their thoughts, each stroke on the keyboard emitted either a high or low pitch tone (the volume and intensity of each tone was held constant), thus subtly associating participant’s positive or negative thoughts with high or low pitch sounds in the environment.

These data revealed that even in this indirect, contextual role, low (vs. high) pitch sounds heard in the background while typing thoughts yielded the same meta-cognitive pattern of effects by polarizing attitudes (see Fig. 5, bottom panel). Once again, the relationship between pitch and thought direction was mediated by thought confidence. Taken together, these studies indicate that changes in pitch reliably influence perceptions of confidence, regardless of whether the pitch originates from the source of the message, the message recipient, or as an unrelated contextual factor in the background. Moreover, changes in pitch not only influence how confident people perceive others to be (when they are talking), but also how confident people perceive themselves to be (when they are talking). Importantly, confidence emerging from pitch can be misattributed to any thought currently in mind, even if those thoughts are totally unrelated to the vocal information. Thus, this confidence is beneficial to persuasion when thoughts are positive but detrimental for persuasion when thoughts are negative.

## Multiple Roles for Vocal Hallmarks of Confidence Beyond Pitch

Although most of our empirical examples have focused on unpacking how the effects of pitch on persuasion are mediated by perceptions of confidence, there are, of course, many other persuasion-relevant vocal qualities. In fact, research has also applied the multiple roles framework described by the ELM to rate of speech, and other aspects of voice linked to confidence such as volume and intonation. These indicators of vocal confidence can also operate as a simple cue for acceptance vs. rejection when thinking is low, and in other roles in other circumstances. For example, an experiment by Chebat et al. (2007) manipulated different properties of a speaker’s voice so that either the intensity (loudness), intonation (variation in pitch), or speech rate (words per minute) was high/low, while the remaining two vocal properties were kept at the speaker’s natural baseline. The topic used in this study (benefits of a new ATM card) was rated as low in personal involvement, suggesting that careful scrutiny of the message was unlikely (i.e., low-elaboration conditions). Attitudes toward the advertisement were more favorable when the speaker sounded high versus low in confidence (i.e., spoke fast vs. slow, with a high vs. low intensity, and when intonation was low vs. high). This suggests that under low elaboration, changes in the nonverbal features of a speakers’ voice influenced persuasion in the same direction implied by the valence of the speaker’s voice (see also Gelinas-Chebat & Chebat, 1992; Miller et al., 1976).

Vocal qualities associated with speaker confidence can also influence persuasion by affecting the valence of thinking. This was illustrated in a study by Chattopadhyay et al. (2003), who employed professional sound technicians to manipulate the speaker’s rate of speech (fast/slow) and vocal pitch (high/low) without affecting other properties of voice. Participants listened to an advertisement delivered by an experienced radio announcer that promoted a health supplement available at local businesses. Importantly, a cover story was explicitly designed to ensure that participants listened carefully to the advertisement (i.e., high elaboration). Following the recording, participants indicated their attitudes toward the product and then wrote down the thoughts they had while listening to the advertisement. Consistent with the idea that vocal hallmarks of confidence can bias the direction of thinking about an issue, participant’s thoughts about the health supplement were significantly more positive when the speaker communicated at a fast (vs. slow) rate of speech. Importantly, thought favorability significantly mediated the relationship between speech rate and attitudes toward the advertisement.

Using the ELM as a theoretical framework, recent research has re-examined the link between rate of speech and persuasion, along with other qualities of voice linked to perceptions of speaker confidence, including vocal intonation (Guyer et al., 2019). These data confirmed that although perceptions of speaker confidence were responsible for the effects of voice on attitudes, the underlying process by which this occurred differed based on whether or not the message recipient was carefully processing the message. In line with the ELM, under high-thinking conditions, perceptions of speaker confidence biased the favorability of thoughts, which in turn served as a guide when forming attitudes toward the topic. In contrast, under low-thinking conditions, speaker confidence did not bias thought-favorability but rather directly influenced attitudes as a peripheral cue.

In addition to biasing the direction of thoughts, vocal confidence linked to changes in rate of speech, intonation, or loudness can also serve as an issue-relevant argument when these changes are informative about the merits of the attitude object under consideration and when elaboration is high. Moreover, recall that vocal confidence can influence the extent to which a recipient processes a persuasive message when thinking is unconstrained. For example, although faster speakers are generally perceived as more confident (Brown et al., 1985; Jiang & Pell, 2014; Scherer et al., 1973), extremely fast speech can reduce a recipient’s ability to process a message, potentially undermining the persuasive benefits of confidence. Indeed, Moore et al. (1986) found that rapid rates of speech were associated with reduced argument quality effects (the relative difference in persuasion between strong and weak arguments) compared to slower rates of speech. In other words, very rapid speech reduced the persuasive impact of strong arguments but increased the persuasive effect of weak arguments. This pattern was replicated in a study by Hausknecht and Moore (1986), and more recently by Guyer et al. (2018b).

Perceived confidence that emerges from vocal hallmarks of confidence can not only decrease but also *increase* thinking under conditions of moderate elaboration. For example, in an experiment by Guyer et al. (2018b), participants heard a speaker with either rising or falling intonation present either strong or weak arguments favoring a policy that required students to work for their university for a minimum of two years in exchange for a reduction in tuition. Next, participants rated the speaker’s confidence and their attitudes toward the proposal. Results showed that falling intonation yielded greater perceived confidence than rising intonation, which increased persuasion by strong arguments and decreased persuasion by weak arguments. Thus, when people’s ability and motivation to think are unconstrained (i.e., moderate elaboration), vocal qualities that influence perceptions of speaker confidence can also influence the success of persuasive communications by affecting the extent to which a recipient thinks about the evidence presented in an advocacy (either by increasing or decreasing thinking).

Finally, communicators with a confident voice can also impact what recipients think about the validity of their thoughts. That is, under high-elaboration conditions, speaker vocal confidence can impact whether or not people use their thoughts by influencing how valid people think their thoughts are—especially when they consider speaker confidence after generating thoughts (Tormala et al., 2007). Finally, as previously stated, if people believe that their judgments are somehow being inappropriately biased by the properties of the communicator’s voice, and they do not want this to occur, they can correct their judgments in a direction opposite to the perceived bias.

As noted, whether the process by which persuasion takes place involves low or high elaboration is important because the consequences associated with those changes are likely to be different. Specifically, research has shown that effortful processing of a message typically yields attitudes that are more accessible, durable over time, resistant to persuasive attacks, and more predictive of behavior than attitudes formed via relatively low-thinking processes (see Petty et al., 1995a, 1995b, for a review). To date, however, no studies have examined these postulated attitude strength outcomes for high versus low confidence speakers. All of these roles for vocal properties, the conditions under which they occur, and their consequences, were summarized in Fig. 2.

## Future Directions

Throughout this review we have described how different vocal hallmarks linked to confidence, whether they originated from the message source, recipient, or even as a background contextual factor, could influence attitudes and persuasion. We also outlined different processes by which this influence could occur (Fig. 2) and provided data where possible throughout our review to show how processes could emerge due to particular moderators.

Although there remain many interesting avenues of inquiry open to further research, one important point to consider is how vocal factors might intersect with cultural considerations. For example, cross-cultural psychologists suggest that power distance (i.e., cultural beliefs that hierarchical power structures are legitimized and acceptable; Hofstede, 1980; Moon et al., 2017) is predominant in some countries (e.g., China) relative to other countries (e.g., Canada, United States). An intriguing possibility is that effects of vocal confidence could be more potent in high power-distance cultures, insofar as such cultures make dominance and subordination primary considerations of social interaction. For example, a message recipient attending to a persuasive message could use vocal confidence cues to determine if the speaker is their superordinate or subordinate, and thus could be more sensitive to perceiving subtle indicators of vocal confidence (i.e., authority), or more responsive to such signals in terms of downstream actions (e.g., showing larger thoughtful biases in favor of confident versus non-confident messages). Through such work, we can extend the generalizability of vocal confidence research, potentially identifying cultural milieus in which this framework has larger (or altered) influences over persuasion.

Another avenue for future research might be to explore the deliberative manipulation of vocal properties (e.g., pitch, speech rate, loudness) by persuasive communicators to elicit feelings of confidence not only in their target audiences but also in themselves. That is, just as communicators modulate their voice intentionally to manage the impressions of others, future studies can benefit from examining to what extent people can also intentionally modulate their own vocal properties to regulate their internal emotional states via bolstering feelings of confidence in desirable thoughts or undermining confidence in unwanted thoughts.

## Footnotes

1 “Multiple roles” is a term used in the context of the Elaboration Likelihood Model (Petty & Cacioppo, 1986a, b) that connotes that variables such as vocal pitch can affect attitudes by different mechanisms in different situations. For a more complete description of each of the five roles a variable can play in affecting attitudes, we refer the reader to the content found under the heading: “Understanding the effects of paralinguistic markers on persuasion: A theoretical framework,” as well as Fig. 2, which provides a visual illustration specifying the antecedents and consequences for each of the multiple roles.

## References

[CR1] Anderson R, Klofstad CA (2012). Preference for leaders with masculine voices holds in the case of feminine leadership roles. PLoS ONE.

[CR2] Andreasen N (1981). Acoustic analysis: An objective measure of affective flattening. Archives of Genetic Psychiatry.

[CR3] Apicella CL, Feinberg DR, Marlowe FW (2007). Voice pitch predicts reproductive success in male hunter-gatherers. Biology Letters.

[CR4] Apple W, Streeter LA, Krauss RM (1979). Effects of pitch and speech rate on personal attributions. Journal of Personality and Social Psychology.

[CR5] Arias P, Rachman L, Liuni M, Aucouturier JJ (2021). Beyond correlation: Acoustic transformation methods for the experimental study of emotional voice and speech. Emotion Review.

[CR6] Aung T, Puts D (2020). Voice pitch: A window into the communication of social power. Current Opinion in Psychology.

[CR7] Babel M, McGuire G, King J (2014). Towards a more nuanced view of vocal attractiveness. PLoS ONE.

[CR8] Bänziger T, Patel S, Scherer KR (2014). The role of perceived voice and speech characteristics in vocal emotion communication. Journal of Nonverbal Behavior.

[CR9] Bollinger D, Greenberg JH, Ferguson CA, Moravcsik EA (1978). Intonation across languages. Universals of human language, phonology.

[CR10] Bond RN, Welkowitz J, Goldschmidt H, Wattenberg S (1987). Vocal frequency and person perception: Effects of perceptual salience and nonverbal sensitivity. Journal of Psycholinguistic Research.

[CR11] Brennan SE, Williams M (1995). The feeling of another′s knowing: Prosody and filled pauses as cues to listeners about the metacognitive states of speakers. Journal of Memory and Language.

[CR12] Briñol P, DeMarree KG (2012). Social metacognition.

[CR13] Briñol P, Petty RE (2009). Source factors in persuasion: A self-validation approach. European Review of Social Psychology.

[CR14] Briñol P, Petty RE, Stavraki M, Lamprinakos G, Wagner B, Díaz D (2018). Affective and cognitive validation of thoughts: An appraisal perspective on anger, disgust, surprise, and awe. Journal of Personality and Social Psychology.

[CR15] Brooke ME, Ng H (1986). Language and social influence in small conversational groups. Journal of Language and Social Psychology.

[CR16] Brown BL, Giles H, Thakerar JN (1985). Speaker evaluations as a function of speech rate, accent and context. Language and Communication.

[CR17] Brown BL, Strong WJ, Rencher AC (1973). Perceptions of personality from speech: Effects of manipulations of acoustical parameters. Journal of the Acoustical Society of America.

[CR18] Cacioppo JT, Petty RE (1982). The need for cognition. Journal of Personality and Social Psychology.

[CR19] Carpenter CJ (2015). A meta-analysis of the ELM’s argument quality X processing type predictions. Human Communication Research.

[CR20] Chaiken S, Maheswaran D (1994). Heuristic processing can bias systematic processing: Effects of source credibility, argument ambiguity, and task importance on attitude judgment. Journal of Personality and Social Psychology.

[CR21] Chattopadhyay A, Dahl DW, Ritchie RJB, Sahin KN (2003). Hearing voices: The Impact of announcer speech characteristics on consumer responses to broadcast advertising. Journal of Consumer Psychology.

[CR22] Chebat J-C, Hedhli KE, Gélinas-Chebat C, Boivin R (2007). Voice and persuasion in a banking telemarketing context. Perceptual and Motor Skills.

[CR23] Cheng JT, Tracy JL, Ho S, Henrich J (2016). Listen, follow me: Dynamic vocal signals of dominance predict emergent social rank in humans. Journal of Experimental Psychology: General.

[CR24] Cowen AS, Elfenbein HA, Laukka P, Keltner D (2019). Mapping 24 emotions conveyed by brief human vocalization. American Psychologist.

[CR25] Dabbs JM, Mallinger A (1999). High testosterone levels predict low voice pitch among men. Personality and Individual Differences.

[CR26] Dunlosky J, Metcalfe J (2009). Metacognition.

[CR27] Evans S, Neave N, Wakelin D, Hamilton C (2008). The relationship between testosterone and vocal frequencies in human males. Physiology & Behavior.

[CR28] Feinberg DR (2008). Are human faces and voices ornaments signaling common underlying cues to mate value?. Evolutionary Anthropology: Issues, News, and Reviews.

[CR29] Feinberg DR, Jones BC, DeBruine LM (2005). The voice and face of woman: One ornament that signals quality?. Evolution and Human Behavior.

[CR30] Feinberg DR, Jones BC, Little AC, Burt DM, Perrett DI (2005). Manipulations of fundamental and formant frequencies influence the attractiveness of human male voices. Animal Behaviour.

[CR31] Fraccaro PJ, Jones BC, Vukovic J, Smith FG, Watkins CD, Feinberg DR, Little AC, Debruine LM (2011). Experimental evidence that women speak in a higher voice pitch to men they find attractive. Journal of Evolutionary Psychology.

[CR32] Gelinas-Chebat C, Chebat JC (1992). Effects of two voice characteristics on the attitudes toward advertising messages. The Journal of Social Psychology.

[CR33] Gelinas-Chebat C, Chebat JC (1999). Impact of voice on source credibility in advertising: A self-monitoring approach. North American Journal of Psychology.

[CR34] Gilbert DT, Osborne RE (1989). Thinking backward: Some curable and incurable consequences of cognitive busyness. Journal of Personality and Social Psychology.

[CR35] Gregory SW, Gallagher TJ (2002). Spectral analysis of candidates’ nonverbal vocal communication: Predicting US presidential election outcomes. Sociology and Psychology Quarterly.

[CR36] Gregory SW, Webster S (1996). A nonverbal signal in voices of interview partners effectively predicts communication accommodation and social status perceptions. Journal of Personality and Social Psychology.

[CR37] Guyer JJ, Briñol P, Petty RE, Horcajo J (2019). Nonverbal behavior of persuasive sources: A multiple process analysis. Journal of Nonverbal Behavior.

[CR38] Guyer, J. J., Briñol, P., Vaughan-Johnston, T. I., Fabrigar, L. R., Moreno, L., Vidal, L., & Petty, R. E. (2020). The role of vocal confidence in persuasion: A self-validation perspective. In *Paper presented at the society for personality and social psychology conference*, in New Orleans, Louisiana, USA.

[CR39] Guyer JJ, Fabrigar LR, Vaughan-Johnston TI (2018). Speech rate, intonation, and pitch: Investigating the bias and cue effects of vocal confidence on persuasion. Personality and Social Psychology Bulletin.

[CR40] Guyer, J. J. Fabrigar, L. R., & Vaughan-Johnston, T. I. (2018c). Vocal confidence and persuasion: How speech rate affects amount of processing as a function of recipient ability and motivation. In *Paper presented at the Ohio State University attitudes conference*, in Columbus, Ohio, USA.

[CR41] Guyer JJ, Fabrigar LR, Vaughan-Johnston TI, Tang C (2017). The counter-intuitive influence of vocal affect on the efficacy of affectively-based persuasive messages. Journal of Experimental Social Psychology.

[CR42] Guyer, J. J., Vaughan-Johnston, T. I., & Fabrigar, L. R. (2018b). Vocal intonation can influence persuasion by moderating the amount of thinking. In *Poster presented at the attitude pre-conference at the Society for Personality and Social Psychology conference*, in Atlanta, Georgia, USA.

[CR43] Halberstadt AG (1983). Family expressiveness styles and nonverbal communication skills. Journal of Nonverbal Behavior.

[CR44] Hall JA (1980). Voice tone and persuasion. Journal of Personality and Social Psychology.

[CR45] Harries MLL, Walker JM, Williams DM, Hawkins S, Hughes IA (1997). Changes in the male voice at puberty. Archives of Disease in Childhood.

[CR46] Harrigan J, Rosenthal R, Scherer K (2008). The new handbook of methods in nonverbal behavior research.

[CR47] Haugtvedt CP, Petty RE (1992). Personality and persuasion: Need for cognition moderates the persistence and resistance of attitude changes. Journal of Personality and Social Psychology.

[CR48] Haugtvedt CP, Strathman A (1990). Situational personal relevance and attitude persistence. Advances in Consumer Research.

[CR49] Hausknecht DR, Moore DL (1986). The effects of time compressed advertising on brand attitude judgments. Advances in Consumer Research.

[CR50] Heesacker M, Petty RE, Cacioppo JT (1983). Field dependence and attitude change: Source credibility can alter persuasion by affecting message-relevant thinking. Journal of Personality.

[CR51] Hodges-Simeon CR, Gaulin SJC, Puts DA (2011). Different vocal parameters predict perceptions of dominance and attractiveness. Human Nature.

[CR52] Hofstede G (1980). Culture’s consequences—International differences in work-related values.

[CR53] Hughes SM, Farley SD, Rhodes BC (2010). Vocal and physiological changes in response to the physical attractiveness of conversational partners. Journal of Nonverbal Behavior.

[CR54] Hughes SM, Mogilski JK, Harrison MA (2014). The perception and parameters of intentional voice manipulation. Journal of Nonverbal Behavior.

[CR55] Jiang, X., & Pell, M. (2014). Encoding and decoding confidence information in speech. In *7th international conference on speech prosody 2014*. 10.21437/speechprosody.2014-102

[CR56] Jiang X, Pell MD (2015). On how the brain decodes vocal cues about speaker confidence. Cortex.

[CR57] Jiang X, Pell MD (2016). Neural responses towards the speaker’s feeling of (un)knowing. Neuropsychologia.

[CR58] Jiang X, Pell MD (2017). The sound of confidence and doubt. Speech Communication.

[CR59] Johnson WF, Ernde RN, Scherer KR, Klinnert MD (1986). Recognition of emotion from vocal cues. Archives of General Psychiatry.

[CR60] Jost JT, Kruglanski AW, Nelson TO (1998). Social metacognition: An expansionist review. Personality and Social Psychology Review.

[CR61] Juslin PN, Laukka P (2003). Communication of emotions in vocal expression and music performance: Different channels, same code?. Psychological Bulletin.

[CR62] Kang YS, Herr PM (2006). Beauty and the beholder: Toward an integrative model of communication source effects. Journal of Consumer Research.

[CR63] Kimble CE, Seidel SD (1991). Vocal signs of confidence. Journal of Nonverbal Behavior.

[CR64] Klofstad CA, Anderson RC, Nowicki S (2015). Perceptions of competence, strength, and age influence voters to select leaders with lower-pitched voices. PLoS ONE.

[CR65] Klofstad CA, Anderson RC, Peters S (2012). Sounds like a winner: Voice pitch influences perception of leadership capacity in both men and women. Proceedings of the Royal Society of London B.

[CR66] Knapp ML, Hall JA, Horgan TG (2014). Nonverbal communication in human interaction.

[CR67] Ko SJ, Sadler MS, Galinsky AD (2015). The sound of power: Conveying and detecting hierarchical rank through voice. Psychological Science.

[CR68] Kreiman J, Sidtis D (2011). Voices and listeners: Toward a model of voice perception. Acoustics Today.

[CR69] Laukka P, Elfenbein HA (2020). Cross-cultural emotion recognition and in-group advantage in vocal expression: A meta-analysis. Emotion Review.

[CR70] Leongómez JD, Binter J, Kubicová L, Stolařová P, Klapilová K, Havlíček J, Roberts SC (2014). Vocal modulation during courtship increases proceptivity even in naive listeners. Evolution and Human Behavior.

[CR71] Leongómez JD, Mileva VR, Little AC, Roberts SC (2017). Perceived differences in social status between speaker and listener affect the speaker's vocal characteristics. PLoS ONE.

[CR72] Lieberman P, Blumstein SE (1988). Speech physiology, speech perception, and acoustic phonetics. Cambridge University Press.

[CR73] Mandal MK (2008). Cultural in-group advantage in accuracy at recognizing vocal expressions of emotion. Psychological Studies.

[CR74] Mazur A, Booth A (1998). Testosterone and dominance in men. Behavioral and Brain Sciences.

[CR75] Mehrabian A, Ferris SR (1967). Inference of attitudes from nonverbal communication in two channels. Journal of Consulting Psychology.

[CR76] Mehrabian A, Williams M (1969). Nonverbal concomitants of perceived and intended persuasiveness. Journal of Personality and Social Psychology.

[CR77] Mello J, Garcia-Marques T, Briñol P, Cancela A, Petty RE (2020). The influence of physical attractiveness on attitude confidence and resistance to change. Journal of Experimental Social Psychology.

[CR78] Meuser W, Nieschlag E (1977). Sexualhormone und Stimmlage des Mannes. DMW - Deutsche Medizinische Wochenschrift.

[CR79] Miller N, Maruyama G, Beaber RJ, Valone K (1976). Speed of speech and persuasion. Journal of Personality and Social Psychology.

[CR80] Monetta L, Cheang HS, Pell MD (2008). Understanding speaker attitudes from prosody by adults with Parkinson’s disease. Journal of Neuropsychology.

[CR81] Moon C, Weick M, Uskul AK (2017). Cultural variation in individuals' responses to incivility by perpetrators of different rank: The mediating role of descriptive and injunctive norms. European Journal of Social Psychology.

[CR82] Moore DL, Hausknecht D, Thamodaran K (1986). Time compression, response opportunity, and persuasion. Journal of Consumer Research.

[CR83] Pedersen MF, Möller S, Krabbe S, Bennett P (1986). Fundamental voice frequency measured by electroglottography during continuous speech. A new exact secondary sex characteristic in boys in puberty. International Journal of Pediatric Otorhinolaryngology.

[CR84] Pell MD (2006). Cerebral mechanisms for understanding emotional prosody in speech. Brain and Language.

[CR85] Pell MD, Monetta L, Paulmann S, Kotz SA (2009). Recognizing emotions in a foreign language. Journal of Nonverbal Behavior.

[CR86] Petty RE, Briñol P, Van Lange PAM, Kruglanski A, Higgins ET (2012). The elaboration likelihood model. Handbook of theories of social psychology.

[CR87] Petty RE, Briñol P, Tormala ZL (2002). Thought confidence as a determinant of persuasion: The self-validation hypothesis. Journal of Personality and Social Psychology.

[CR88] Petty RE, Briñol P, Tormala ZL, Wegener DT, Kruglanski AW, Higgins ET (2007). The role of meta-cognition in social judgment. Social psychology: Handbook of basic principles.

[CR89] Petty RE, Cacioppo JT (1979). Issue involvement can increase or decrease persuasion by enhancing message-relevant cognitive responses. Journal of Personality and Social Psychology.

[CR90] Petty RE, Cacioppo JT, Berkowitz L (1986). The elaboration likelihood model of persuasion. Advances in experimental social psychology.

[CR91] Petty RE, Cacioppo JT (1986). Communication and persuasion: Central and peripheral routes to attitude change.

[CR92] Petty RE, Cacioppo JT, Schumann D (1983). Central and peripheral routes to advertising effectiveness: The moderating role of involvement. Journal of Consumer Research.

[CR93] Petty RE, Harkins SG, Williams KD (1980). The effects of group diffusion of cognitive effort on attitudes: An information processing view. Journal of Personality and Social Psychology.

[CR94] Petty RE, Haugtvedt CP, Smith SM, Petty RE, Krosnick JA (1995). Elaboration as a determinant of attitude strength: Creating attitudes that are persistent, resistant, and predictive of behavior. Attitude strength: Antecedents and consequences.

[CR95] Petty RE, Krosnick J (1995). Attitude strength: Antecedents and consequences.

[CR96] Petty RE, Schumann DW, Richman SA, Strathman AJ (1993). Positive mood and persuasion: Different roles for affect under high and low elaboration conditions. Journal of Personality and Social Psychology.

[CR97] Petty RE, Wegener DT (1993). Flexible correction processes in social judgment: Correcting for context-induced contrast. Journal of Experimental Social Psychology.

[CR98] Petty RE, Wegener DT, Gilbert D, Fiske S, Lindzey G (1998). Attitude change: Multiple roles for persuasion variables. The handbook of social psychology.

[CR99] Petty RE, Wegener DT, White PH (1998). Flexible correction processes in social judgment: Implications for persuasion. Social Cognition.

[CR100] Petty RE, Wells GL, Brock TC (1976). Distraction can enhance or reduce yielding to propaganda: Thought disruption versus effort justification. Journal of Personality and Social Psychology.

[CR101] Pisanski K, Bryant GA, Eidsheim NS, Meizel KL (2019). The evolution of voice perception. The oxford handbook of voice studies.

[CR102] Pisanski K, Feinberg DR, Frühholz S, Belin P (2019). Vocal attractiveness. Oxford handbook of voice perception.

[CR103] Pisanski K, Oleszkiewicz A, Plachetka J, Gmiterek M, Reby D (2018). Voice pitch modulation in human mate choice. Proceedings of the Royal Society B.

[CR104] Pittam J, Gallois C (1987). Predicting impressions of speakers from voice quality: Acoustic and perceptual measures. Journal of Language and Social Psychology.

[CR105] Puts DA (2016). Human sexual selection. Current Opinion in Psychology.

[CR106] Puts DA, Apicella CL, Cárdenas RA (2012). Masculine voices signal men's threat potential in forager and industrial societies. Proceedings of the Royal Society B: Biological Sciences.

[CR107] Puts DA, Gaulin SJC, Verdolini K (2006). Dominance and the evolution of sexual dimorphism in human voice pitch. Evolution and Human Behavior.

[CR108] Puts DA, Hodges C, Cárdenas RA, Gaulin SJC (2007). Men’s voices as dominance signals: Vocal fundamental and formant frequencies influence dominance attributions among men. Evolution and Human Behavior.

[CR109] Rucker DD, Tormala ZL, Petty RE, Briñol P (2014). Consumer conviction and commitment: An appraisal-based framework for attitude certainty. Journal of Consumer Psychology.

[CR110] Schaal B, Tremblay RE, Soussignan R, Susman EJ (1996). Male testosterone linked to high social dominance but low physical aggression in early adolescence. Journal of the American Academy of Child & Adolescent Psychiatry.

[CR111] Scherer KR (1988). Facets of emotions: Recent research. Erlbaum.

[CR112] Scherer KR, Frühholz S, Belin P (2019). Acoustic patterning of emotion vocalizations. Oxford handbook of voice perception.

[CR113] Scherer KR, Banse R, Wallbott HG (2001). Emotion inferences from vocal expression correlate across languages and cultures. Journal of Cross-Cultural Psychology.

[CR114] Scherer KR, Clark-Polner E, Mortillaro M (2011). In the eye of the beholder? Universality and cultural specificity in the expression and perception of emotion. International Journal of Psychology.

[CR115] Scherer KR, London H, Wolf JJ (1973). The voice of confidence: Paralinguistic cues and audience evaluation. Journal of Research in Personality.

[CR116] Schroeder J, Epley N (2015). The sound of intellect: Speech reveals a thoughtful mind, increasing a job candidate’s appeal. Psychological Science.

[CR117] Schroeder J, Epley N (2016). Mistaking minds and machines: How speech affects dehumanization and anthropomorphism. Journal of Experimental Psychology: General.

[CR118] Sell A, Bryant GA, Cosmides L, Tooby J, Sznycer D, von Rueden C, Krauss A, Gurven M (2010). Adaptations in humans for assessing physical strength from the voice. Proceedings of the Royal Society of London B, Biological Sciences.

[CR119] Simon-Thomas ER, Keltner DJ, Sauter D, Sinicropi-Yao L, Abramson A (2009). The voice conveys specific emotions: Evidence from vocal burst displays. Emotion.

[CR120] Smith SM, Shaffer DR (1991). Celebrity and cajolery: Rapid speech may promote or inhibit persuasion through its impact on message elaboration. Personality and Social Psychology Bulletin.

[CR121] Smith SM, Shaffer DR (1995). Speed of speech and persuasion: Evidence for multiple effects. Personality and Social Psychology Bulletin.

[CR122] Smith VL, Clark HH (1993). On the course of answering questions. Journal of Memory and Language.

[CR123] Sorokowski P, Puts D, Johnson J, Żółkiewicz O, Oleszkiewicz A, Sorokowska A, Kowal M, Borkowska B, Pisanski K (2019). Voice of authority: Professionals lower their vocal frequencies when giving expert advice. Journal of Nonverbal Behavior.

[CR124] Swaddle JP, Reierson GW (2002). Testosterone increases perceived dominance but not attractiveness in human males. Proceedings of the Royal Society of London. Series B: Biological Sciences.

[CR125] Taylor AM, Reby D (2010). The contribution of source-filter theory to mammal vocal communication research: Advances in vocal communication research. Journal of Zoology.

[CR126] Tigue CC, Borak DJ, O’Connor JJM, Schandl C, Feinberg DR (2012). Voice pitch influences voting behavior. Evolution and Human Behavior.

[CR127] Titze IR (1994). Principles of voice production.

[CR128] Tormala ZL, Briñol P, Petty RE (2007). Multiple roles for source credibility under high elaboration: It’s all in the timing. Social Cognition.

[CR129] Tremblay RE, Schaal B, Boulerice B, Arseneault L, Soussignan R, Pérusse D (1997). Male physical aggression, social dominance and testosterone levels at puberty. Biosocial Bases of Violence.

[CR130] Van Zant AB, Berger J (2020). How the voice persuades. Journal of Personality and Social Psychology.

[CR131] Vaughan-Johnston, T. I.., Guyer, J. J., Fabrigar, L. R., & Briñol, P. (2020). Vocal confidence can increase persuasion as an argument. In *Paper accepted at the**Midwestern Psychological Association* conference, in Chicago, Illinois, USA. (Conference cancelled).

[CR132] Wegener DT, Petty RE, Zanna MP (1997). The flexible correction model: The role of naive theories of bias in bias correction. Advances in experimental social psychology.

[CR133] Wolff SE, Puts DA (2010). Vocal masculinity is a robust dominance signal in men. Behavioral Ecology and Sociobiology.

[CR134] Zuckerman M, Miyake K (1993). The attractive voice: What makes it so?. Journal of Nonverbal Behavior.

